# Suicide-related outcomes in veterans with post-traumatic headache: a retrospective cohort study

**DOI:** 10.1016/j.lana.2025.101299

**Published:** 2025-11-13

**Authors:** Sarah E. Anthony, Manali A. Phadke, Richard B. Lipton, Daniel G. Rogers, Lisa A. Brenner, John P. Ney, Hamada H. Altalib, X. Michelle Androulakis, Amy S. Grinberg, Melissa Skanderson, Hung-Mo Lin, Joel D. Scholten, Brenda T. Fenton, Elizabeth K. Seng, Jason J. Sico

**Affiliations:** a VA Headache Centers of Excellence (HCoE) Research, Evaluation, Education, and Engagement Activities Center for Headache (RE3ACH), Orange, CT, USA; b Department of Neurology, Yale School of Medicine, New Haven, CT, USA; c Yale Center for Analytical Sciences, Yale University, New Haven, CT, USA; d Yale School of Public Health, New Haven, CT, USA; e Montefiore Headache Center, Department of Neurology, Albert Einstein College of Medicine, Bronx, NY, USA; f Saul R. Korey Department of Neurology, Albert Einstein College of Medicine, Bronx, NY, USA; g Pain Research, Informatics, Multi-morbidities, and Education Center, VA Connecticut Healthcare System, West Haven, CT, USA; h Department of Psychiatry, Yale School of Medicine, New Haven, CT, USA; i Department of Physical Medicine and Rehabilitation, Psychiatry, and Neurology, University of Colorado, Anschutz Medical Campus, Aurora, USA; j VA Brain Health Coordinating Center, Rocky Mountain Regional Veterans Affairs Medical Center, Eastern Colorado Health Care System, Aurora, USA; k VA Connecticut Healthcare System, West Haven, CT, USA; l Ralph H Johnson VA Healthcare System and Medical University of South Carolina, Charleston, SC, USA; m Department of Anesthesiology, Yale School of Medicine, New Haven, CT, USA; n Physical Medicine & Rehabilitation Program Office, Veterans Health Administration, Washington, DC, USA; o Ferkauf Graduate School of Psychology, Yeshiva University, Bronx, NY, USA; p Department of Internal Medicine, Yale School of Medicine, New Haven, CT, USA

**Keywords:** Headache, Traumatic brain injury, Suicide, Health Policy

## Abstract

**Background:**

Post-traumatic headache (PTH) is a common sequela of traumatic brain injury (TBI). Although there is a known association between TBI and suicide risk in veterans, the association between PTH and suicide-related outcomes in veterans with TBI is relatively unknown. We aimed to evaluate the association between PTH and suicide-related outcomes in veterans compared to matched controls diagnosed with TBI but no history of headaches.

**Methods:**

This retrospective cohort study was conducted with Veterans Health Administration and Department of Defense electronic health record data from fiscal years 2008 through 2020. Veterans with PTH were matched to a control group who had TBI and no headache disorders. Relative risk was estimated using propensity score-weighted log-binomial models that evaluated differences in suicidal ideation, suicide attempts, and suicide death.

**Findings:**

Of the 95,224 veterans included in the total sample, 85,730 were male (90.0%) and 9,949 were female (10.0%). The average age of the sample was 45.9 years (SD = 16.6). 73,500 (77.2%) were White, Non-Hispanic, 17,256 (18.1%) were Black, Non-Hispanic, and 4,468 (4.7%) were classified as other or mixed race. Of the 47,612 veterans diagnosed with PTH, 4,618 (9.7%) reported suicidal ideation or suicide attempts compared to 3,162 (6.6%) in the control group. Veterans with PTH had increased risk of suicidal ideation (RR, 1.45; 95% CI, 1.39–1.51) and suicide attempts (RR, 1.66; 95% CI, 1.50–1.83) compared to matched controls. Using inverse probability weighting to adjust for confounding, these results remained significant. When adjusting for potential confounders, as well as prior suicidal ideation or suicide attempts, there was no significant difference in risk of suicide death in veterans with PTH (RR, 0.83; 95% CI, 0.67–1.02) compared to those with TBI without headache.

**Interpretation:**

Veterans with PTH have an increased risk of suicidal ideation and suicide attempts compared to veterans with TBI and without headache. There was no difference in suicide mortality between the two groups. Clinicians should be aware of heightened suicide risk among veterans with PTH and be especially diligent in terms of screening for suicide risk and related medical and mental health comorbidities that contribute to increased risk.

**Funding:**

This study was supported by the United States 10.13039/100000738Department of Veterans Affairs special purpose medical service funding (SP80DPE.1-0160).

**Research in context**

**Evidence before this study**

This study builds on prior research that has established the burden of post-traumatic headache (PTH) as a common sequelae of traumatic brain injury (TBI), the association between chronic headache disorders and suicide risk, and the high prevalence of suicide among veterans with TBI. Despite a robust literature search using many online databases such as PubMed, Web of Science, etc., there is a lack of research examining PTH and its association with suicide-related outcomes. This retrospective cohort study was conducted using Veterans Health Administration (VHA) and Department of Defense (DOD) electronic health records from fiscal year 2008 through fiscal year 2020. Inclusion criteria were based on *ICD-CM* codes validated by neurologists who were board-certified in headache medicine and prior chart reviews conducted by an independent external peer review process team. Comorbidities known to influence suicide risk were also considered.

**Added value of this study**

This study is among the largest to evaluate the risk of suicide-related outcomes in veterans with PTH compared to matched controls with TBI and without headache disorders. Key findings include:

• Veterans with PTH had a significantly higher risk of suicidal ideation (RR, 1.45; 95% CI, 1.39–1.51) and suicide attempts (RR, 1.66; 95% CI, 1.50–1.83).
• These associations remained significant even after adjusting for common medical and psychiatric comorbidities.
• Among Veterans, PTH was not significantly associated with increased risk of suicide death.

This study highlights the need for targeted suicide prevention strategies in veterans with PTH. These findings suggest that PTH should be considered a distinct risk factor for suicide-related thoughts and behaviors.

**Implications of all the available evidence**

These findings reinforce the importance of identifying and screening for suicide risk among veterans with PTH, integrating suicide prevention strategies into headache and TBI management programs, researching neurobiological mechanisms linking PTH and suicide risk, and conducting longitudinal studies examining how PTH influences suicide-related outcomes over time. This study underscores the need for continued clinical vigilance and policy interventions to mitigate suicide risk amongst members of this at-risk veteran population.

## Introduction

Worldwide, chronic headache disorders are one of the top causes of years lived with disability^1^; thereby significantly impacting quality of life for millions. In the United States (US), this burden is pronounced among veterans with a history of traumatic brain injury (TBI),^2^ the “signature wound” of recent conflicts in Iraq and Afghanistan. Between 2000 and 2020, 434,618 military service members sustained a TBI.^3^ One of the most common sequelae of TBI is post-traumatic headache (PTH).^2^^,^^4^ From fiscal years (FY) 2008–2020, 51,755 veterans were diagnosed with PTH within the VHA Headache Cohort.^5^ Despite advances toward identifying comorbidities and risk factors, PTH remains a complex challenge to treat due to the heterogeneity of symptoms and phenotypes and the preponderance of medical and mental health comorbidities.^6^

Over the last two decades, suicide has remained a pressing public health concern in the US among members of the veteran population. According to the 2020 National Veteran Suicide Prevention Annual Report, veterans exhibited higher rates of suicide compared to non-veteran US adults, even after adjusting for population differences in age and sex.^7^ The Department of Veterans Affairs (VA) has prioritized suicide prevention and implemented a comprehensive suicide risk assessment for veterans receiving care within VHA, including those with TBI. Multiple studies have shown that a history of TBI increases the risk of suicide.^8^, ^9^, ^10^ Additional research has shown that veterans with chronic headache disorders are at higher suicide risk.^11^^,^^12^ No study to date has specifically explored the extent to which PTH is associated with suicide-related outcomes in veterans compared to veterans with a history of TBI and without headache. The current study evaluated differences in suicide-related outcomes, which included suicidal ideation (SI), suicide attempts (SA), and suicide death, among veterans with PTH and veterans with TBI and without headache. We hypothesized that veterans with PTH would have a higher risk of suicide-related outcomes compared to matched veterans with TBI and without headache, and that these findings would persist when controlling for baseline medical and mental health comorbidities.

## Methods

### Study design and data sources

This retrospective cohort study evaluated differences in suicide-related outcomes among veterans with PTH and non-headache matched controls with TBI during FY 2008–2020. In the VHA, this corresponded to October 1, 2007, through September 30, 2020. An adapted validated algorithm identified veterans with headache disorders diagnosed with an *International Classification of Diseases Clinical Modification* (*ICD-CM)* code by a VHA provider from electronic VHA clinical and administrative data sources,^5^^,^^13^ and inclusion and exclusion criteria were applied to identify the final VHA Headache Cohort. Data extraction for this study from the VHA Headache Cohort included all veterans with at least one outpatient visit in the VHA for a headache diagnosis from FY 2008–2020.

To determine each distinct diagnosis, *ICD-CM* codes were considered to the first decimal (Supplementary Table S1). *ICD-CM* codes were used to identify veterans with headache diagnoses and suicide-related outcomes. Entry into the cohort occurred on the date of the first encounter with a coded headache disorder within the study period. The VHA Headache Cohort protocol has been approved by the VA Connecticut Healthcare System Institutional Review Board. This study followed the Strengthening the Reporting of Observational Studies in Epidemiology (STROBE) reporting guidelines for cohort studies.

### Participants

#### PTH cases

**Fig. 1:**
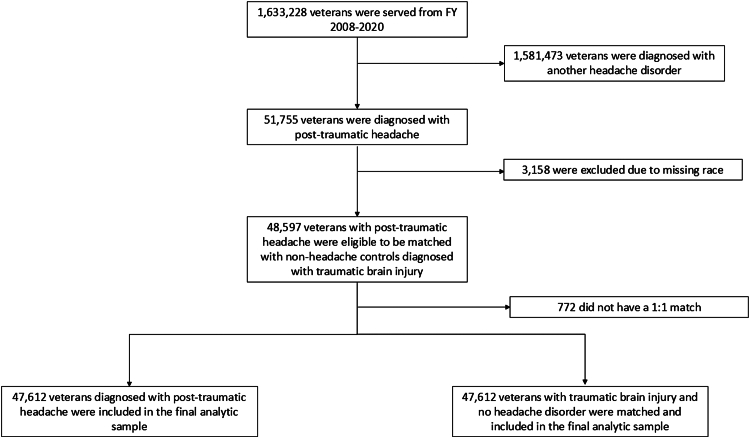
**Flowchart of veterans with post-traumatic headache and non-headache matched controls with traumatic brain Jury included in the final analytic sample**.

To construct the PTH group, this study used the VHA Headache Cohort.^5^ The sampling timeframe spanned the period during which the VHA used both the *ICD-*9-CM and *ICD-*10-CM; a group of four neurologists board-certified in headache medicine independently reviewed a Centers for Medicare and Medicaid Services General Equivalence Mapping crosswalk linking *ICD-*9-CM headache codes to the *ICD-*10-CM codes and reached consensus on all headache diagnoses, including those for PTH.^14^ For the current study, only patients with a healthcare provider's diagnostic code for PTH were included (Supplementary Table S1). In a preliminary chart review study designed to validate headache diagnosis codes in the VHA electronic health record, 323 charts of veterans with *ICD-CM* PTH codes in FY 2017 were abstracted by an independent external peer review process team, which included registered nurses, registered health information administrators, and registered health information technicians.^15^ When comparing the independent clinical impression to the *ICD-CM* diagnosis code, the majority (248/323, 76.8%) had a definite clinical impression of PTH. Most of the other charts had a clinical impression consistent with a PTH phenotype (e.g., either migraine or tension-type headache, n = 36/323, 11.1%) or a broad clinical impression of “headache” (35/323, 10.8%); these data provide evidence for the validity of the use of the *ICD-CM* codes for PTH to identify veterans with post-traumatic headache in the VHA. Given the overlap of phenotypes, PTH cases with another documented headache type were not excluded, which is consistent with the International Classification of Headache Disorders, 3rd Edition (ICHD-3). According to the ICHD-3, trauma or injury to the head and/or neck needs to precede the headache to be diagnosed with PTH.^16^ For the purposes of the analyses, it was assumed that all veterans with PTH had sustained a TBI prior to headache diagnosis. PTH cases who were unable to be matched, including those missing any demographic variables used to match, were excluded from the final sample (Fig. 1).

#### Matched cases with TBI and without headache

Veterans with a PTH diagnosis were matched to the control group with TBI and without headache. Both groups were matched on age (ordinal variable in 5-year increments), FY, Veterans Integrated Services Network (VISN, geographically defined care delivery networks), sex, race, and ethnicity, which were extracted at the date of entry into the cohort. The matched control group needed to have one outpatient visit within the same FY as the corresponding PTH case. The index date for controls was a randomly selected outpatient visit within the same FY as the corresponding PTH case. TBI diagnoses were identified from three sources that included either an *ICD-CM* code from the Department of Defense (DoD) and VA Informatics and Computing Infrastructure (DAVINCI), an *ICD-CM* code from VHA's Corporate Data Warehouse (CDW) database, or a Health Factors positive screen from the CDW database. FY was included to account for policy implementations^7^ and the *ICD-*9-CM to *ICD-*10-CM transition.^15^ Documented TBIs occurred before and after active-duty service. Matched controls with a positive TBI diagnosis were excluded if they had any headache diagnosis.

### Outcomes of interest: suicide-related outcomes

Suicide-related outcomes included SI, SA, SI or SA, and suicide death. All suicide-related outcomes were identified with *ICD-CM* codes during the study period, starting up to 30 days before the index date or headache diagnosis date. The date range of suicide deaths was extended past the study period until the end of FY 2020. Suicide death was obtained from the VA and Department of Defense Mortality Data Repository. Suicide death was determined by identifying the National Death Index (NDI) records containing suicide-related *ICD-*10-CM codes as the underlying cause of death (Supplementary Table S1). Death certificate data from the NDI remains the gold standard for US mortality data.^9^

#### Covariates

Medical and psychiatric conditions associated with PTH^17^, ^18^, ^19^ and suicide-related outcomes^7^^,^^20^, ^21^, ^22^ were taken into consideration. Covariates were extracted at baseline, defined as the period from one year before to 6 months after the index date. Age was evaluated continuously, and FY was assessed categorically, taken at the cohort's entry date. Diagnoses of epilepsy, major depressive disorder (MDD), post-traumatic stress disorder (PTSD), insomnia, alcohol use disorder, and drug use disorder were categorical. They were extracted from VHA administrative data sources using validated algorithms.^13^^,^^23^ Psychiatric and substance use comorbidities required diagnoses during at least one inpatient or two outpatient visits within the study period. The Charlson Comorbidity Index (CCI) was continuous and used to measure medical comorbidities based on *ICD-CM* codes found in administrative data. Non-headache pain was identified from Kaiser pain groups based on *ICD-CM* codes from VHA's CDW database that included abdominal, back, limb, muscular, chest, neuropathy, temporomandibular disorders, fibromyalgia, pelvic, and systemic pain.

### Statistical analysis

All study variables were described above, for veterans with diagnosed PTH and for the matched controls with TBI and without headache. Categorical variables are described using frequencies and percentages. Continuous variables are displayed using medians (interquartile range [IQR]) for non-normally distributed variables and means (standard deviations) for normally distributed variables. Differences between the PTH group and matched controls were evaluated using the chi-square (χ2) test for categorical variables, and the Student's T-test or Wilcoxon rank-sum test for continuous variables.

After matching, both unadjusted and adjusted models were used to estimate the relative risk of suicide-related outcomes in patients with PTH compared with their matched controls. Conventional propensity score methods typically require estimating scores using the entire target sample; however, the presence of millions of veterans in the control group, combined with the complexity of administrative data and current computational constraints, rendered this approach infeasible. Therefore, we adopted a retrospective cohort design in which each PTH individual was matched to a randomly selected control from the same stratum, defined by unique combinations of key baseline characteristics (age category, FY, VISN, sex, race, and ethnicity). To further account for residual baseline imbalances, we conducted inverse propensity score–weighted outcome analyses. The probability of having PTH was estimated using a logistic regression model that included established predictors of suicide risk: age, non-headache pain, insomnia, epilepsy, Charlson Comorbidity Index (CCI), post-traumatic stress disorder (PTSD), major depressive disorder (MDD), alcohol use disorders, and drug use disorders.

By matching, our causal inference targeted the population of individuals with PTH, and our analysis aimed to estimate the effect of PTH exposure on suicide-related outcomes had the PTH-exposed individuals not been exposed. To achieve this, inverse probability of treatment weights (IPTW) were calculated for veterans in the matched sample: PTH cases were assigned a weight of 1, and controls were weighted by the risk of having PTH (i.e., the ratio of their predicted probability of PTH exposure to the probability of non-exposure). To reduce the influence of extreme weights, we applied trimming at the 1st and 99th percentiles. Application of the propensity score weights led to substantial improvement in covariate balance, with all standardized mean differences (SMDs) reduced to below 0.1, supporting adequate balance between PTH cases and matched controls (Supplementary Figure S1). As a sensitivity analysis, a multivariable quasi-Poisson model was also fitted using the same set of covariates as those in the propensity score model to assess the robustness of our findings (Supplementary Table S2). For the suicide death model, SI and SA were included as covariates, allowing for estimation of the direct effect of PTH on suicide death. For both groups, SI and SA had to be present within 30 days before the index date through the end of the study period (FY 2020). Suicide death was extended past the study period until December 31, 2020. All analyses were two-tailed with alpha set at 0.05 and were performed using SAS version 9.4 (Cary, NC).

### Role of the funding source

The funder of the study had no role in study design, data collection, data analysis, data interpretation, or writing of the report.

## Results

From FY 2008–2020, the VHA served 1,633,228 unique veterans with headache, of which 51,755 veterans were diagnosed with PTH during the study period. 3,158 were excluded due to missing race. An additional 772 did not have a 1:1 match. The final analytic sample had 47,612 PTH cases and 47,612 matched TBI cases without headache (Fig. 1). The sample mostly consisted of White (77.2%) males (90.0%) with an average age of 45.9 (SD = 16.6) years. All baseline medical and mental health comorbidities examined were significantly higher among those with PTH compared to controls, including non-headache pain (73.2% vs 42.4%; *p* < 0.001) and PTSD (48.0% vs 26.7%; *p* < 0.001; Table 1). A similar trend was seen in SI (9.2% vs 6.3%; *p* < 0.001) and SA (2.2% vs 1.3%; *p* < 0.001) but not in suicide death (0.3% vs 0.3%; *p* = 0.78; Table 2). Of those with a recorded suicide death from the NDI, 88 (27%) were flagged with a prior SI or SA (Table 2). When subset to those who had a suicide-related outcome, all baseline medical and mental health comorbidities differed slightly in the PTH cases and control group (Supplementary Table S3).

**Table 1:** Baseline sociodemographic and health characteristics among veterans with post-traumatic headache and veterans with traumatic brain injury without headache.^a^

Baseline sociodemographic characteristics	PTH cases (n = 47,612)	TBI without headache cases (n = 47,612)	Total (n = 95,224)
Age, mean, (SD), yr	45.94 (16.6)	45.94 (16.7)	45.94 (16.6)
Sex, No. (%)			
Male	42,865 (90.0)	42,865 (90.0)	85,730 (90.0)
Female	4,747 (10.0)	4,747 (10.0)	9,494 (10.0)
Race, No. (%)			
White/Caucasian	36,750 (77.2)	36,750 (77.2)	73,500 (77.2)
Black/African American	8,628 (18.1)	8,628 (18.1)	17,256 (18.1)
Asian	638 (1.3)	638 (1.3)	1,276 (1.3)
Mixed race	567 (1.2)	567 (1.2)	1,134 (1.2)
Native American	527 (1.1)	527 (1.1)	1,054 (1.1)
Pacific Islander	502 (1.1)	502 (1.1)	1,004 (1.1)
Ethnicity, No. (%)			
Hispanic	4,141 (8.7)	4,141 (8.7)	8,282 (8.7)
Year of diagnosis, No. (%)			
2008	8 (0.02)	8 (0.02)	16 (0.02)
2009	1,697 (3.6)	1,697 (3.6)	3,394 (3.6)
2010	1,951 (4.1)	1,951 (4.1)	3,902 (4.1)
2011	2,121 (4.5)	2,121 (4.5)	4,242 (4.5)
2012	2,589 (5.4)	2,589 (5.4)	5,178 (5.4)
2013	3,119 (6.6)	3,119 (6.6)	6,238 (6.6)
2014	3,769 (7.9)	3,769 (7.9)	7,538 (7.9)
2015	3,583 (7.5)	3,583 (7.5)	7,166 (7.5)
2016	8,446 (17.7)	8,446 (17.7)	16,892 (17.7)
2017	5,992 (12.6)	5,992 (12.6)	11,984 (12.6)
2018	5,451 (11.5)	5,451 (11.5)	10,902 (11.5)
2019	4,919 (10.3)	4,919 (10.3)	9,838 (10.3)
2020	3,967 (8.3)	3,967 (8.3)	7,934 (8.3)
Baseline health characteristics, No. (%)^b^			
Non-headache pain	34,845 (73.2)	20,168 (42.4)	55,013 (57.8)
PTSD	22,868 (48.0)	12,730 (26.7)	35,598 (37.4)
MDD	12,354 (25.6)	6,733 (14.1)	19,087 (20.0)
Insomnia	6,428 (13.5)	2,360 (5.0)	8,788 (9.2)
Alcohol use disorder	5,808 (12.2)	4,828 (10.1)	10,636 (11.2)
Drug use disorder	4,544 (9.5)	3,502 (7.4)	8,046 (8.5)
Epilepsy	2,548 (5.4)	1,811 (3.8)	4,359 (4.6)
CCI, mean (SD)	0.70 (1.6)	0.51 (1.4)	0.60 (1.5)

Abbreviations: PTH, Post Traumatic Headache; SD, standard deviation; PTSD, posttraumatic stress disorder; MDD, major depressive disorder; CCI, Charlson Comorbidity Index; VA, Veterans Affairs.

a PTH Cases and TBI without Headache (i.e., matched Control cases) are matched on age (±5 year window), sex, race/ethnicity, Veterans Integrated Services Network, and fiscal year.

b For all baseline health characteristics, *p* < 0.001.

**Table 2:** Suicide-related outcomes among veterans with post-traumatic headache and veterans with traumatic brain injury without headache.^a^

Suicide-related outcomes^b^	PTH cases (n = 47,612)	TBI without headache cases (n = 47,612)	Total (n = 95,224)
Suicide ideation, No. (%)	4,361 (9.2)	3,012 (6.3)	7,373 (7.4)
Suicide attempt, No. (%)	1,046 (2.2)	631 (1.3)	1,677 (1.8)
Suicide death, No. (%)	158 (0.3)	163 (0.3)	321 (0.3)

Abbreviations: PTH, Post Traumatic Headache; VA, Veterans Affairs.

a PTH Cases and TBI without Headache (i.e., matched control cases) are matched on age (±5 year window), sex, race/ethnicity, Veterans Integrated Services Network, and fiscal year.

b For suicide ideation and suicide attempt *P* < 0.001. For suicide death, P = 0.78.

**Fig. 2:**
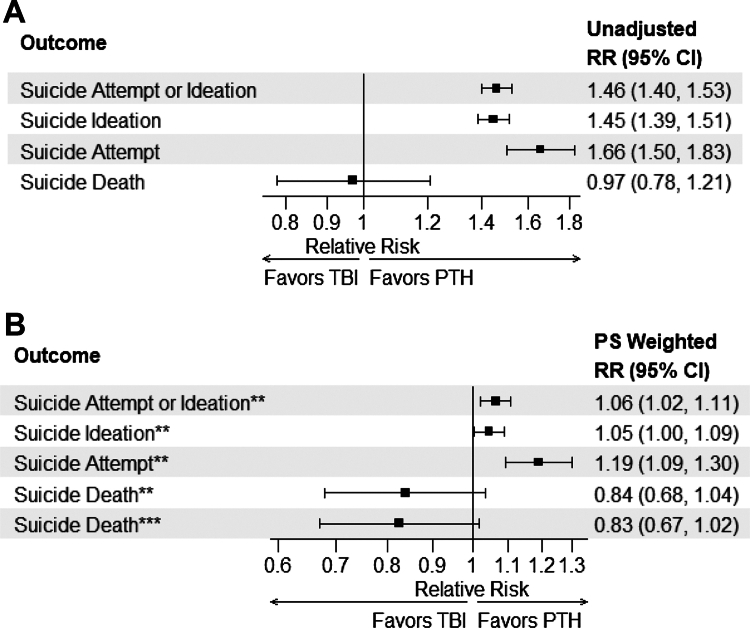
**Association between post-traumatic headache (vs Matched Controls∗) and suicide-related outcomes**. ∗Matched Controls included those with a documented traumatic brain injury (TBI) and excluded any headache diagnosis. Post-traumatic headache (PTH) and Controls are matched on age (±5 year window), sex, race/ethnicity, Veterans Integrated Services Network, and fiscal year. (A) Forest plot shows the unadjusted effect of PTH vs TBI on suicide-related outcomes. (B) Forest plot shows the propensity score weighted effect of PTH vs TBI on suicide-related outcomes. ∗∗Inverse probability treatment weighting: Weights were calculated as the inverse of the propensity of having PTH, with the propensity estimated using logistic regression including age, non-headache pain, PTSD, MDD, insomnia, epilepsy, alcohol use disorder, drug use disorder, and Charlson Comorbidity Index as predictors. ∗∗∗Models were weighted as above and further adjusted for suicidal ideation and suicide attempt.

PTH patients had increased unadjusted risk of SI [(RR, 1.45; 95% CI, 1.39–1.51) *p* < 0.0001], SA [(RR, 1.66; 95% CI, 1.50–1.83) *p* < 0.0001], and combined SI or SA [(RR, 1.46; 95% CI 1.40–1.53) *p* < 0.0001], compared to veterans with TBI and without headache. Although reduced, the IPTW-adjusted effect of PTH on suicide-related outcomes remained statistically significant for SI [(RR, 1.05; 95% CI, 1.00, 1.09) *p* = 0.03], SA [(RR, 1.19; 95% CI 1.09–1.30) *p* = 0.001], and combined SI or SA [(RR, 1.06; 95% CI 1.02–1.11) *p* = 0.002]. PTH was not associated with an increased risk of suicide mortality in any model with or without adjustment (Fig. 2). Notably, the sensitivity analysis based on conventional covariate adjustment produced results closely aligned with those from the IPTW method (Supplementary Table S2).

## Discussion

While TBI, migraine, and chronic headache are individually associated with increased rates of suicide related behaviors,^24^ previous works have not considered an incremental effect of PTH on the risk of suicide-related outcomes above that of TBI. This cohort study has three key findings: (1) the burden of medical and mental health comorbidities known to impair quality of life and increase suicide risk were significantly higher among those with PTH; (2) while much of the variance for the difference in SI and SA is accounted for by these comorbidities, after adjusting for these factors, PTH was independently associated with a 5% increased risk of SI and a 19% increased risk of SA, and; (3) additionally adjusting for SI and SA, PTH status was not associated with suicide death in any model.

Reasons for the heightened risk of SI and SA among veterans with PTH and respective matched controls examined in this sample are unclear, though differences in the neuropathophysiology may contribute. Studies investigating hypothalamic connectivity networks have found differences in static and dynamic functional network connectivity (FNC) between those with mTBI and PTH compared to those with mTBI without PTH.^25^, ^26^, ^27^ A prospective neuroimaging study recruited TBI patients with PTH and mTBI patients without PTH and examined whole brain features focusing on the FNC states and other temporal properties. Their findings showed differences in static FNC (sFNC) and dynamic FNC (dFNC), patients with mTBI and PTH showed specific abnormalities in sFNC.^25^ Other functional connectivity studies that have examined FNC in regions containing the hypothalamus report decreased functional connectivity in brain areas involved in pain processing, which can exacerbate emotional distress and underlying mental health issues, further increasing the risk of ideation and attempts.^26^ Furthermore, another neuroimaging study examined differences in brain structure between those with PTH and migraine and found differences in regional volumes, cortical thickness, surface area, and brain curvature, suggesting that PTH is a distinct headache diagnosis and should be treated as such.^27^ Future research conducting comparative studies that follow patients with PTH and migraine over time can help delineate the natural progression of each disease and identify unique biomarkers.

While PTH and migraine differ in their etiology and patterns of altered brain connectivity,^27^ they share several underlying mechanisms, including trigeminovascular activation and neuroinflammation, which influence pain modulation.^4^ Nearly three-quarters (73.2%) of veterans with PTH in this sample had another non-headache pain diagnosis compared with just 42% of the headache-free control group. In the VHA, veterans receiving care for migraine, the most common phenotype of PTH, often have high levels of concurrent non-headache pain disorders.^28^^,^^29^ In this study, physical pain, along with other psychiatric comorbidities, was shown to be associated with elevated SI and SA in veterans, which is consistent with evidence previously published.^30^^,^^31^ Pain management among this group should be continuous and use a multimodal approach to address not only the physical sensation, but also psychosocial factors that influence pain perception.

### Limitations

Among the limitations, this study used administrative data, which brings an array of ascertainment challenges.^29^ Although administrative data allowed us to identify patterns and analyze a large unique patient sample, the administrative data only captured encounters reported within the VHA and may contain diagnostic and documentation errors. Only 88 (27%) of those with a suicide death had a documented SI or SA event. However, suicide screening within the VHA occurs primarily through an annual suicide screen, which boasts high completion rates,^7^ further underlining the limitation of using an *ICD-CM* only approach. Alternatively, this may be related to a confluence of factors related to documentation, assignment of a diagnosis where a history was shared by the veteran (without accompanying medical records), variability in diagnoses among different clinicians, or missing events due to treatment by outside providers (e.g., car accidents or non-military related injuries). SI has been known to be underreported,^32^ as it falls under other diagnostic criteria for psychiatric disorders such as MDD and bipolar disorder. When additionally accounting for MDD, only 143 (45%) of those who died of suicide had a coded SI or SA. Additional work to improve patient care and comprehensive documentation regarding suicide-related thoughts and behaviors includes comparing screeners (e.g., Columbia- Suicide Severity Risk Scale Screener) and suicide-related *ICD-CM* codes to further differentiate between identifying missing or incomplete diagnoses.

Although the association between veterans with PTH vs matched controls and suicide death was not statistically significant, it approached significance (*p* = 0.08) with more independent suicide deaths observed in the control group compared to the PTH group. While we adjusted for many known confounders, particularly those related to psychiatric and neurological disorders, we acknowledge the possibility of residual confounding from unmeasured factors such as healthcare utilization or TBI severity. To quantify how strongly an unmeasured confounder would need to relate to both PTH and suicide-related outcomes to negate our findings, we calculated E-values. Across all outcomes, E-values for the propensity score weighted model ranged from 1.26 to 1.72. These values fall in the moderate range relative to our model's effect estimates, indicating that while residual confounding remains possible, a confounder of this magnitude is unlikely given known risk factors (Supplementary Table S4). Furthermore, our analyses showed that PTH was significantly associated with an increased risk of suicide attempt and suicidal ideation. The same data indicated that suicide attempts and suicidal ideation were, in turn, associated with an increased risk of suicide-related death (data not shown). These findings suggest that the effect of PTH on suicide-related death is likely mediated through this pathway rather than occurring via a direct effect. Future longitudinal studies with follow-up are needed to characterize this pathway and to determine whether targeted interventions for suicidal ideation and attempt in veterans with PTH can reduce subsequent suicide mortality.

The comparison between veterans with PTH vs TBI matched controls without headache may not fully isolate the unique contribution of PTH to suicide-related outcomes. Future research should consider incorporating additional comparison groups to better differentiate the effect of PTH. We expect the change in the direction of the association to stem from the difference in healthcare utilization between PTH cases and matched controls. Higher engagement with healthcare services increases the opportunities for screening, early detection of suicide risk, and timely intervention. These protective factors could explain the lower number of suicide deaths, even after adjustment. However, healthcare utilization could not be directly examined in this model as these visits would not be captured longitudinally. The statistical analysis only examined medical and psychiatric covariates at baseline. Baseline covariates were identified up to one year before and six months after PTH diagnosis or match date, without accounting for medical or psychiatric conditions that could have emerged later. While the time after baseline is important to assess suicide risk, not restricting the time period for the selected covariates will introduce biases, as PTH itself could affect the likelihood of developing MDD, PTSD, etc. Also, the delay in PTH diagnosis in veterans relative to headache onset suggests that the higher baseline rates seen in the PTH group may only represent the start of increasingly divergent rates of comorbidities between the groups. The baseline covariates still provide important control for risk factors present before the full onset of the condition, mitigating the likelihood of biased results based on when medical or psychiatric conditions emerged. In future research, a time-to-event analysis with time-varying covariates can be explored, capturing the evolving nature of comorbid conditions between PTH diagnosis and suicide-related outcomes, as well as accounting for potential censoring. As for the scope of this study, the baseline covariates help ensure a clearer understanding of pre-existing risk factors when comparing the PTH and matched controls.

Another limitation concerns the gap in accruing comorbidities; the control group was extracted from the time of TBI diagnosis, while the PTH group was extracted from the time of diagnosis, potentially several months post-TBI, giving the latter more time to develop TBI-related conditions. During this time, veterans with TBI who could potentially develop PTH but did not survive to be diagnosed, could account for differences seen in our model with suicide death. Moreover, our analyses did not examine TBI severity, as this could further elucidate the risk of suicide; however, ascertainment challenges surrounding the documentation of TBI continue to be widespread.^33^ Lastly, these findings may not generalize to other healthcare systems or populations. Differences between the veteran and civilian population include TBI mechanisms, access to care, and demographic differences: (1) Veterans often sustain deployment-related TBIs caused by blast injuries,^2^ which may differ from TBIs in civilians, such as those from sport-related impacts; (2) Veterans receive healthcare within an integrated system like the VHA, which may provide consistent access to mental health and suicide prevention resources; (3) The veteran population predominantly consists of males (90.0%) who are less likely to report suicide ideation^34^ and are more likely to have access to firearms.^35^

### Conclusion

To date, this is one of the largest PTH cohort studies in the country to examine the risk of suicide. These findings suggest that veterans diagnosed with PTH merit close monitoring for SI and SA. Pain-related diagnoses, substance use disorders, and mental health comorbidities emerged as important factors related to suicide risk in veterans with PTH. Beyond these common risk factors, negative emotions experienced during stressful periods of readjustment into civilian life (e.g., unable to reintegrate into their communities)^36^ can lead to feelings of perceived burdensomeness, which has been implicated in veterans with a higher risk for suicide.^37^^,^^38^ VHA initiatives aim to reduce veteran suicides by implementing universal screening and clinically indicated evaluation procedures.^39^^,^^40^ Expanded focus on suicide prevention and access to centralized resources are beneficial to veterans at risk and continue to inform national policies.

## Contributors

S.E. Anthony was involved in the literature search, study design and concept, data interpretation, and writing & editing of the original draft. M.A. Phadke was involved in data analysis, data interpretation, and provided critical approval of the manuscript. R.B. Lipton, H.H. Altalib, and X. M. Androulakis were involved in the study concept and design, data interpretation, and provided critical approval of the manuscript. D.G. Rogers, L. Brenner, A.S. Grinberg, J. Ney, H.M. Lin, and J.D. Sholten were involved in the data interpretation and provided critical approval of the manuscript. M. Skanderson was involved in data collection, data interpretation, and validation. E. Seng and B. Fenton were involved in the study design and concept, data collection, data interpretation, supervision, and provided critical approval of the manuscript. J. Sico was involved in the study design and concept, data interpretation, funding acquisition, supervision, and provided critical approval of the manuscript. All authors had access to all the data and responsibility for the decision to submit the paper.

## Data sharing statement

Due to VA regulations and our ethics agreements, data sets used in this study are not permitted to leave the VA firewall without a Data Use Agreement. VA data are made freely available to researchers with an approved VA study protocol. For more information, please visit https://www.virec.research.va.gov or contact the VA Information Resources Center at VIReC@va.gov.

## Declaration of interests

Dr. Lipton has received research support from the National Institutes of Health, NIA, SOL, NINDS, and Alzheimer's Association. He serves as consultant, advisory board member, or has received honoraria or research support from AbbVie/Allergan, Axon, Axsome, Biohaven, Clexio, Eli Lilly, Grifols, Lundbeck, Manistee, Pfizer, Satsuma, Shiratronics, Teva, and Tonix. He receives royalties from *Wolff's Headache*,7th and 8th edition (Oxford University Press, 2009), Wiley, and Informa. He holds stock/options in Axon, Biohaven, CoolTech, Manistee, and Wizermed. Dr. Seng has consulted for GlaxoSmithKline, Pfizer, and Abbvie and served as an advisory board member for Abbvie; All other authors report no disclosures relevant to the manuscript.
