# Supplementary material for: Suicide-related outcomes in veterans with post-traumatic headache: a retrospective cohort study
Source: Lancet Reg Health Am. 2025 Nov 13;53:101299. doi: 10.1016/j.lana.2025.101299 (PMC12662118; doi:10.1016/j.lana.2025.101299)
Supplement: Supplementary Figure and Tables [file mmc1.pdf]

Supplementary Table 1. International Classification of Diseases Clinical Modification (ICD-CM) Versions 9 and 10 Codes Included Exposure and Main Outcomes

Diagnosis	ICD-9-CM Code	ICD-10-CM Code
**Traumatic Brain Injury**	310.x, 800.x, 801.x, 803.x, 804.x, 850.x, 851.x, 852.x, 853.x, 907.0x, V15.5x, V15.52x, V15.59x	DOD.0101x, DOD.0102x, DOD0103x, DOD.0105x, F07.81x, S04.04x, S06.x, Z87.82x
**Post-Traumatic Headache**	339.20, 339.21, 339.22	G44.301, G44.309, G44.311, G44.319, G44.321, G44.329
**Suicide Attempt**	E950.x, E951.x, E953.x, E958.x, E959.x	T14.xx, T36.xx, T37.xx, T38.xx, T39.xx, T40.xx, T41.xx, T42.xx, T43.xx, T44.xx, T45.xx, T46.xx, T47.xx, T48.xx, T49.xx, T50.xx, T51.xx, T52.xx, T53.xx, T54.xx, T55.xx, T56.xx, T57.xx, T58.xx, T59.xx, T60.xx, T61.xx, T62.xx, T63.xx, T64.xx, T65.xx, T71.xx, X71.xx, X72.xx, X73.xx, X74.xx, X75.xx, X76.xx, X77.xx, X78.xx, X79.xx, X80.xx, X81.xx, X82.xx, X83.xx,

**Suicidal Ideation**	V62.84	R45.850, R45.851
**Suicide Death**	N/A	X60-X84, U03, Y87.0

List of International Classification of Diseases (ICD-9 and ICD-10) codes used to define the exposure, outcome, and comorbid conditions in this study.

Supplementary Table 2. Association Between Post-Traumatic Headache (vs. Matched Controls)[*] and Suicide-Related Outcomes

Suicide-Related Outcomes	Adjusted[**] RR (95%CI) *N*=95,224	Adjusted[***] RR (95%CI) *N*=95,224
Suicidal Ideation or Suicide Attempt	1.08 (1.03-1.13)	N/A
Suicidal Ideation	1.07 (1.02-1.12)	N/A
Suicide Attempt	1.17 (1.05-1.30)	N/A
Suicide Death	0.82 (0.65-1.03)	0.81 (0.64-1.02)

[*]Matched Controls included those with a documented TBI and excluded any headache diagnosis. PTH and Controls are matched on age (±5 year window), sex, race/ethnicity, Veterans Integrated Services Network, and fiscal year.

[**]Adjusted for age, fiscal year, non-headache pain, PTSD, MDD, insomnia, epilepsy, Alcohol Use Disorder, Drug Use Disorder, and Charlson Comorbidity Index

[***]Adjusted for the same covariates as well as suicide ideation and suicide attempt

4

5

Supplementary Table 3. Baseline Sociodemographic and Health Characteristics Among Veterans with Suicide-Related Outcomes*			
**Characteristic**	**PTH Cases (n=4,729)**	**TBI without Headache Cases (n=3,284)**	**Total (n= 8,013)**
Age, mean, (SD), yr	42.13 (14.5)	40.23 (14.3)	41.35 (14.4)
Sex, No. (%)			
Male	4,286 (90.6)	3,035 (92.4)	7,321 (91.4)
Female	443 (9.4)	249 (7.6)	692 (8.6)
Race, No. (%)			
White/ Caucasian	3,626 (76.7)	2,536 (77.2)	6,162 (76.9)
Black/African American	885 (18.7)	598 (18.2)	1,483 (18.5)
Asian	58 (1.2)	32 (1.0)	90 (1.1)
Mixed race	48 (1.0)	40 (1.2)	88 (1.1)
Native American	59 (1.3)	42 (1.3)	101 (1.3)
Pacific Islander	53 (1.1)	36 (1.1)	89 (1.1)
Ethnicity, No. (%)			
Hispanic	408 (8.6)	299 (9.1)	707 (8.8)
Year of diagnosis, No. (%)			
2008	1 (0.02)	1 (0.03)	2 (0.02)
2009	268 (5.7)	166 (5.1)	434 (5.4)
2010	266 (5.6)	191 (5.8)	457 (5.7)
2011	318 (6.7)	213 (6.5)	531 (6.6)

2012	388 (8.2)	258 (7.9)	646 (8.1)
2013	419 (8.9)	326 (9.9)	745 (9.3)
2014	437 (9.2)	301 (9.2)	738 (9.2)
2015	373 (7.9)	310 (9.4)	683 (8.5)
2016	829 (17.5)	560 (17.1)	1,389 (17.3)
2017	537 (11.4)	360 (11.0)	897 (11.2)
2018	429 (9.1)	268 (8.2)	697 (8.7)
2019	305 (6.5)	197 (6.0)	502 (6.3)
2020	159 (3.4)	133 (4.1)	292 (3.6)
Baseline Health Characteristics, No. (%)			
Non-headache pain**	3,777 (79.9)	1,760 (53.6)	5,537 (69.1)
PTSD**	3,183 (67.3)	1,591 (48.5)	4,774 (59.6)
MDD**	2,174 (46.0)	1,119 (34.1)	3,293 (41.1)
Insomnia**	921 (19.5)	370 (11.3)	1,291 (16.1)
Alcohol Use Disorder	1,591 (33.6)	1,097 (33.4)	2,688 (33.6)
Drug Use Disorder	1,504 (31.8)	1,047 (31.9)	2,551 (31.8)
Epilepsy**	478 (10.1)	214 (6.5)	692 (8.6)
CCI, mean (SD)**	0.76 (1.6)	0.53 (1.4)	0.67 (1.5)

Abbreviations: PTH, Post Traumatic Headache; SD, standard deviation; PTSD, posttraumatic stress disorder; MDD, major depressive disorder; CCI, Charlson Comorbidity Index; VA, Veterans Affairs

* PTH Cases and TBI without Headache (i.e., matched Control cases) are matched on age (± 5 year window), sex, race/ethnicity, Veterans Integrated Services Network, and fiscal year.

**For baseline health characteristics, $P < .001$

Supplementary Figure 1. Standardized Mean Differences (SMD) Before and After Propensity Score Weight

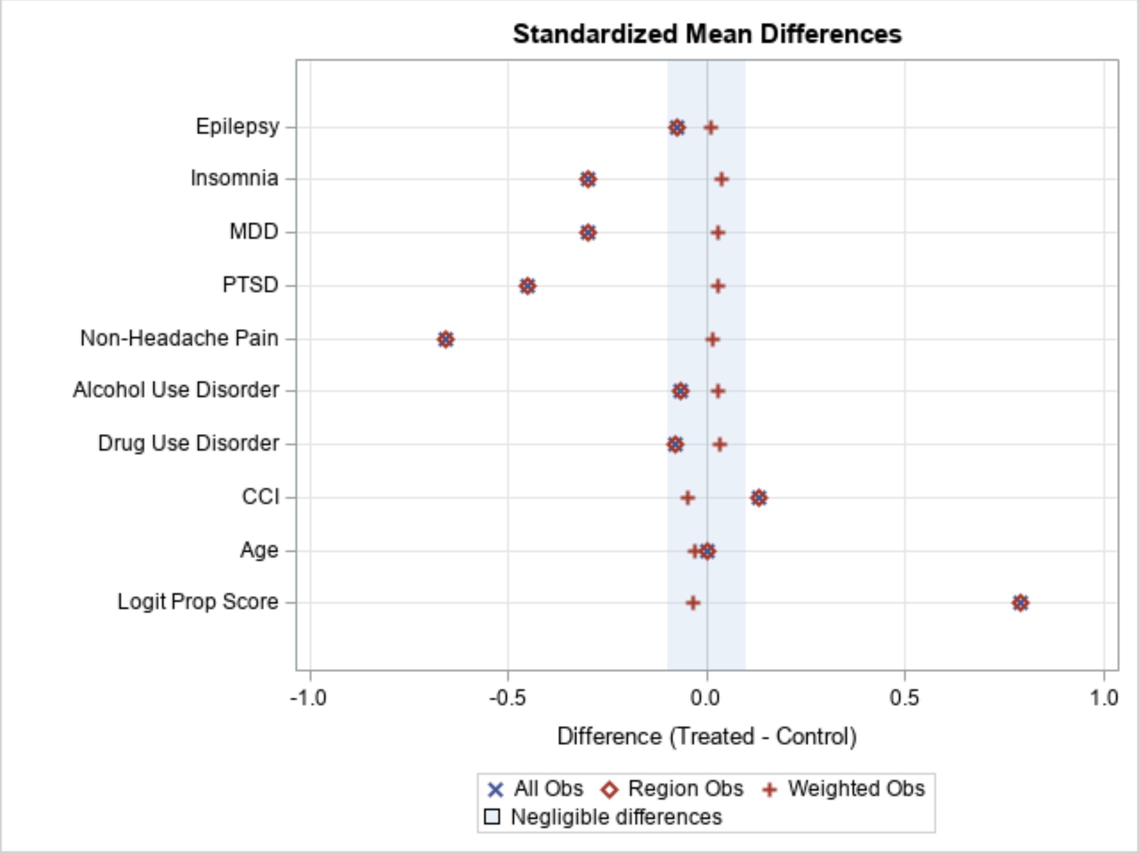

Plot showing standardized mean differences (SMDs) between the post-traumatic headache (PTH) cases vs. TBI matched controls before and after inverse probability of treatment weighting (IPTW). Covariates included age, Charlson Comorbidity Index (CCI), drug use disorder, alcohol use disorder, non-headache pain, PTSD, MDD, insomnia, and epilepsy. The shaded region denotes the range of negligible differences (SMD < 0.1). After weighting, covariate balance improved with most SMDs falling within the acceptable threshold, indicating good balance between groups.

**Supplementary Table 4. E-values for Suicide-Related Outcomes**		
**Suicide-Related Outcome**	**Propensity Score Weighted E-value**	**Covariate Adjusted E-value**
Suicidal Ideation or Suicide Attempt	1.32	1.37
Suicide Attempt	1.67	1.61
Suicidal Ideation	1.26	1.34
Suicide Mortality	1.67	1.75
Suicide Mortality*	1.72	1.78
*adjusting for covariates as well as suicidal ideation and suicide attempt		
